# Beyond Single-Score Matching: A Two-Dimensional Propensity Score Method for Mixed Covariate Types

**DOI:** 10.21203/rs.3.rs-10284547/v1

**Published:** 2026-08-03

**Authors:** Kostiantyn Botnar, Justin T. Nguyen, Kamil Khanipov, George Golovko

**Affiliations:** The University of Texas Medical Branch at Galveston

**Keywords:** Propensity score matching, covariate balance, machine learning, electronic health records, observational studies

## Abstract

Propensity score matching (PSM) reduces confounding bias in observational studies, yet traditional single-score approaches assume linear covariate-treatment relationships. This assumption often fails when applied to complex electronic health record data that contain mixed categorical and continuous variables and nonlinear interactions. We introduce two-dimensional PSM (2D-PSM), which estimates separate propensity scores for categorical and numerical confounders and then performs matching in two-dimensional space under an elliptical caliper constraint. We compared 2D-PSM with traditional single-score PSM using five machine learning classifiers across 5 real-world (RW) clinical datasets (n = 460 – 61,926) and 21 synthetic datasets (n = 10,000) with systematically varied confounder complexity and categorical-to-continuous ratios. Matching employed 1:1 greedy nearest-neighbor algorithm with adaptive calipers (0.1× to 0.5× propensity score interquartile range). Balance was assessed using univariate and multivariate metrics. In multivariate assessment and the smallest caliper (0.1), 2D-PSM performed comparably to conventional PSM on RW datasets and consistently outperformed it in synthetic data experiments. With larger calipers, 2D-PSM outperformed single-score methods in 85% of datasets. Our 2D-PSM approach provides superior multivariate balance for datasets with heterogeneous covariate types and complex interactions, particularly with moderate calipers.

## Introduction

Randomized clinical trials remain the gold standard for investigating treatment effects, yet conducting them with numerous controlled confounders may not always be feasible due to ethical concerns, high costs, or time constraints (Rosenbaum and Rubin 1983; Chen et al. 2022). Consequently, medical research increasingly relies on retrospective observational studies that leverage electronic health records (EHRs), which lack randomized treatment assignments but offer larger sample sizes and greater diversity in patient populations. To address confounding bias in observational studies, propensity score matching (PSM) has been widely adopted across epidemiology, medicine, and public health research (Rosenbaum and Rubin 1983; Austin et al. 2021; Guo et al. 2020; Rosenbaum and Rubin 1985). PSM creates balanced distributions of confounders between treatment and control groups by matching subjects based on their estimated probability of receiving treatment (Rosenbaum and Rubin 1983; Rosenbaum and Rubin 1985).

Logistic regression (LR) remains the most commonly utilized method for estimating propensity scores (PSs) (Kane et al. 2020; Guo et al. 2020; Cannas and Arpino 2019; Setoguchi et al. 2008; Lee et al. 2010; Langworthy et al. 2023). However, LR's effectiveness depends critically on the correct specification of the model, including appropriate functional forms for continuous variables and relevant interaction terms. Misspecification of the LR model may fail to achieve adequate cohort balancing, particularly when relationships between covariates and treatment assignment are nonlinear or involve complex interactions (Cannas and Arpino 2019; Lee et al. 2010; Drake 1993).

To address these limitations, machine learning (ML) techniques have emerged as alternatives for PS estimation. Classification and regression trees (CART) (Cannas and Arpino 2019; Setoguchi et al. 2008; Lee et al. 2010; D’Agostino 1998), Random Forest (RF) (Zhao et al. 2016), Support Vector Machines, and gradient boosting methods (Tu 2019) have been proposed to extract relationships between treatment assignment and predictors without assuming specific parametric forms. These approaches can potentially capture nonlinear relationships and interactions automatically, improving covariate balance and reducing bias (Lee et al. 2010). Studies using simulated data have demonstrated that ensemble methods such as boosted CART and RF can outperform traditional logistic regression in certain scenarios, particularly when the true PS model is complex (Setoguchi et al. 2008; Lee et al. 2010; McCaffrey et al. 2004). McCaffrey et al. (2004) found advantages of using gradient-boosted regression over LR for PS estimation by capturing nonlinearities, whereas Cannas and Arpino (2019) showed that LR, RF, and neural networks performed robustly compared with boosted CARTs and naïve Bayes. More recently, Super Learner, an ensemble method that selects optimal models via cross-validation, has demonstrated lower bias than LR on both simulated and real-world (RW) EHR data (Pirracchio and Carone 2018; Neugebauer et al. 2016; Gharibzadeh et al. 2018).

Despite these methodological advances, evaluations of PSM approaches have predominantly relied on synthetic datasets with homogeneous covariate structures (Cannas and Arpino 2019; Drake 1993; Franchetti 2022). Moreover, both traditional LR and ML-based approaches share a fundamental characteristic: they estimate a single PS by combining all covariates into one prediction model. This single-score approach compresses all covariate information—regardless of whether covariates are categorical or continuous, binary or multi-level—into a unidimensional summary measure used for matching.

EHR data present a particularly challenging environment for PSM due to the heterogeneous nature of clinical covariates. Healthcare datasets typically contain a mixture of categorical variables (sex, race, ethnicity, disease status) and continuous variables (age, body mass index, laboratory values), each with distinct distributional properties and potentially different relationships with treatment assignment. Categorical variables exhibit discrete distributions with limited support, while continuous variables span broader ranges with more nuanced variation. Furthermore, the mechanisms by which these different covariate types influence treatment decisions may differ fundamentally: categorical factors often represent eligibility criteria or practice patterns, whereas continuous measurements may exhibit threshold effects or nonlinear dose-response relationships.

The conventional single-score PSM approach compresses this heterogeneous information into a single scalar PS, potentially leading to differential balance across covariate types. Covariate selection and PS model specification involve inherent trade-offs in balancing different covariates, particularly when those covariates have varying theoretical importance or associations with treatment assignment (Franklin et al. 2014; Austin 2009). When covariates differ substantially in their relationships to treatment assignment, achieving balance across all covariates simultaneously may be challenging, as matching prioritizes overall PS similarity over dimension-specific balance (Rosenbaum and Rubin 1984; Rassen et al. 2012). This is particularly relevant in clinical studies, where both categorical and continuous covariates are important confounders that require adequate balance.

Recent work has highlighted that covariate balance should be assessed not only using univariate metrics such as the absolute standardized mean difference (ASMD) for continuous variables and the standardized difference in proportions (SDP) for categorical variables, but also using multivariate measures that account for the joint distribution of covariates (Lee et al. 2010; Iacus et al. 2011; Gu and Rosenbaum 1993). Metrics such as L1 distance (Iacus et al. 2011) and Mahalanobis Balance (MB) (Gu and Rosenbaum 1993; Rosenbaum and Rubin 1984) have been proposed as alternatives that capture overall distributional similarity. However, even these multivariate metrics may not fully address the fundamental challenge posed by mixed covariate types when matching occurs in a single-dimensional PS space.

To address the limitations of single-score PSM for datasets with mixed covariate types, we propose a novel two-dimensional PSM (2D-PSM) approach. Our method estimates separate PSs for categorical and numerical confounders and then performs matching in a two-dimensional space under an elliptical caliper constraint. This decomposition explicitly accounts for the heterogeneous nature of clinical data and enables dimension-specific balance optimization. By treating categorical and continuous covariates as distinct dimensions in the matching process, 2D-PSM can achieve balanced cohorts that satisfy balance criteria across both covariate types simultaneously.

In this study, we evaluate the performance of 2D-PSM against traditional single-score PSM on systematically designed synthetic datasets with known treatment-assignment mechanisms and varying levels of complexity. We employed multiple ML classifiers for PS estimation in both single-score and two-dimensional frameworks.

## Methods

### Datasets

#### Real World Datasets.

To evaluate 2D-PSM performance on authentic clinical data, we analyzed five RW deidentified EHR datasets derived from separate research projects, representing diverse clinical contexts and covariate structures. The RW dataset 1 (RWD1) (n = 22,905) included patients with end-stage renal disease and 18 covariates, including extensive comorbidity indicators. The RW dataset 2 (RWD2) (n = 13,911) comprised female burn patients receiving estrogen therapy and included only 3 demographic covariates (Song et al. 2025). The RW dataset 3 (RWD3) (n = 61,926) comprised reconstructive surgery cases for burn-wound patients, with 7 covariates and balanced treatment prevalence. The RW dataset 4 (RWD4) (n = 460) comprised patients with pulmonary embolism, with 14 mostly categorical covariates and a high treatment prevalence. The RW dataset 5 (RWD5) (n = 809) included obese transplant recipients with 5 covariates (Dongur et al. 2024). These datasets span sample sizes from 460 to 61,926 patients, treatment prevalences from 1.3% to 78.3%, and covariate compositions from 3 to 18 covariates, providing a comprehensive evaluation of 2D-PSM across clinically relevant scenarios that differ in statistical power, covariate dimensionality, and covariate type composition (Table 1).

#### Synthetic Data Generation.

We generated 21 synthetic datasets to systematically evaluate 2D-PSM under controlled conditions, with known treatment-assignment mechanisms. Each dataset comprised 10,000 observations with 20 covariates, a binary treatment indicator, and the true PS. The synthetic data were generated using a factorial design with treatment-assignment complexity (7 scenarios) and covariate composition (3 variants), yielding 21 unique datasets (7 × 3). The three covariate composition variants used numerical-to-binary proportions of 10:10 (balanced), 5:15 (more binary), and 15:5 (more numerical).

Numerical covariates were generated from four distributions cycling through standard normal (μ = 0, σ = 1), lognormal (μ = 0, σ = 0.5), uniform (−2 to 2), and exponential (λ = 1). Binary covariates were generated from Bernoulli distributions with prevalences ranging from 0.08 to 0.50. All 20 covariates were randomly shuffled to eliminate ordering effects, and numerical covariates were standardized (μ = 0, σ = 1).

Treatment assignment coefficients used an 80/20 ratio of predictor to noise variables (applied separately to numerical and binary covariates). Predictor coefficients were half strong (∣β∣ ∈ [0.85, 1.1]) and half moderate (∣β∣ [0.4, 0.6]) with balanced signs. The seven scenarios (A-G) represented increasing complexity (Table 2). PSs were calculated using logistic regression with Gaussian noise (σ = 0.5) added to the linear predictor, which was then clipped to [0.02, 0.98], with the intercept adjusted to achieve ~ 30% treatment prevalence.

### Two-Dimensional PSM

#### PS Estimation.

Traditional PSM estimates a single score by modeling treatment assignment as a function of all covariates simultaneously. Our 2D-PSM approach decomposes the covariate space into categorical and numerical dimensions and estimates separate PS for each dimension.

For each dataset, categorical covariates were encoded as 0/1 for binary variables, whereas multilevel categorical variables (e.g., race and ethnicity) were one-hot encoded. All numerical covariate values were standardized. We used multiple ML algorithms to evaluate the robustness of 2D-PSM across various modeling approaches. The models employed for estimating PSs included Bernoulli Naïve Bayes (BNB), Logistic Regression (LR), Linear Support Vector Machine (LSVM), Random Forest (RF), and XGBoost (XGB). Both dimensions used the same models, except that the BNB model was excluded from the numerical one. Consequently, each categorical-numerical model combination resulted in a dataset containing the record ID and the PS values derived from categorical and numerical features.

#### Two-Dimensional Matching Algorithm.

Matching in 2D PS space was performed using a greedy nearest-neighbor algorithm without replacement. The algorithm processed treated individuals sequentially (in random order to avoid ordering bias), matching each to the nearest available control individual in 2D space while respecting the elliptical caliper constraint. The elliptical caliper was defined as (1).

(1) $${\left(\frac{|PS_{ct}-PS_{cc}|}{C_{c}}\right)}^{2}+{\left(\frac{|PS_{nt}-PS_{nc}|}{C_{n}}\right)}^{2}\leq 1\;,$$

where PS_ct_ and PS_cc_ – PS of treated and control cohorts in the categorical space, respectively, PS_nt_ and PS_nc_ – PS of treated and control cohorts in the numerical space, respectively, C_c_ and C_n_ – calipers for categorical and numerical dimensions, respectively.

We employed adaptive calipers calculated as a fraction of the interquartile range (IQR) of PS in each dimension. We tested four caliper widths: 0.1 × IQR, 0.2 × IQR, 0.3 × IQR, and 0.5 × IQR, calculated separately for categorical and numerical dimensions. The nearest neighbor was selected using Euclidean distance in 2D PS space. For neighbor selection, we employed a KD-Tree spatial index with k = 100 nearest neighbors per treated individual. Both strategies enforced matching without replacement, with treated individuals randomly shuffled to minimize ordering bias.

### Evaluation

#### Univariate Balance Assessment.

We assessed covariate balance before and after matching using established metrics appropriate for each covariate type. For categorical covariates, we calculated the SDP (Franklin et al. 2014; Austin 2009), with values below 0.1 indicating acceptable balance. For continuous covariates, we calculated the ASMD (Franklin et al. 2014; Austin 2009) and the Variance Ratio (VR) (Franklin et al. 2014). ASMD values below 0.1 typically indicate an acceptable mean balance, while VR values close to 1.0 indicate balanced variance. We then computed the percentage improvement in balance, as shown in (2).

$$BI=\frac{M_{before}-M_{after}}{M_{before}}•\;100$$

where BI – balance improvement, M_before_ and M_after_ – metric values before and after PSM, respectively.

For VR, improvement was calculated as in (3) to account for deviations from the ideal value of 1.0.

$$BI=\left(1-\frac{|1-VR_{after}|}{|1-VR_{before}|}\right)•100$$

where BI – balance improvement, VR_before_ and VR_after_ – variation ratio values before and after PSM, respectively.

#### Multivariate Balance Assessment.

To assess balance across multiple covariates simultaneously, we calculated MB between treatment and control cohorts in multidimensional covariate space. MB accounts for correlations among covariates through the inverse covariance matrix, with lower values indicating better overall balance. Categorical covariates were one-hot encoded and combined with continuous covariates to form a complete covariate matrix. The BI was calculated as the percentage improvement after matching.

To contextualize 2D-PSM performance, we conducted analyses using traditional (one-dimensional) PSM with all covariates, employing the same ML models as in 2D-PSM. Categorical covariates were one-hot encoded, and all features were combined into a single covariate matrix. The balance assessment used the same univariate and multivariate metrics described above, enabling direct comparison between 2D-PSM and traditional PSM. For each dataset and caliper width, we identified the approach that achieved the best balance.

### Software tools

All analyses were implemented in Python 3.10 using NumPy (2.1.2), pandas (2.2.3), scikit-learn (1.7.0), xgboost (2.1.4), and SciPy (1.15.1). All analyses used a random seed of 42 for reproducibility.

## Results

We evaluated 2D-PSM against the traditional approach across 26 datasets (5 EHR and 21 synthetic) that represent diverse levels of complexity and covariate structures. For each dataset, we tested four caliper widths (0.1×, 0.2×, 0.3×, and 0.5× IQR), yielding 104 unique experimental conditions.

### Multivariate Balance Assessment

Overall, 2D-PSM achieved superior or equivalent multivariate BI in 73.1% of datasets when using the most stringent caliper (0.1×IQR), increasing to 80.2% at moderate calipers (0.2× and 0.3×IQR, respectively), and 88.5% with the largest caliper (0.5×IQR) (Table 3). These findings demonstrate that the two-dimensional approach provides robust advantages for datasets containing mixed covariate types, with performance gains becoming more pronounced as matching constraints are relaxed.

The 2D-PSM approach consistently improved MB compared with traditional PSM across all caliper values. The average increase in BI ranged from 2.8% at the most restrictive caliper (0.1) to 7.5% at the largest caliper (0.5), with maximum improvements of 7%, 12%, 18%, and 26% for calipers 0.1, 0.2, 0.3, and 0.5, respectively. Overall, the 2D-PSM method achieved high MB values across all conditions, with an average BI of 95.5% (SD: 3.5%) for caliper 0.1, 94.0% (SD: 5.2%) for caliper 0.2, 93.7% (SD: 6.0%) for caliper 0.3, and 93.5% (SD: 3.5%) for caliper 0.5. These findings indicate that while tighter calipers yield marginally higher BI with lower variability, larger calipers benefit more substantially from the 2D-PSM enhancement, suggesting that the method is particularly effective at recovering balance in scenarios where conventional PSM matching is more prone to covariate imbalance.

Our method consistently achieved similar coverage of the treated cohort across different caliper values, with about 70–73% of treated patients matched to controls on average. The highest coverage occurred at caliper 0.2 (73.2%, SD = 17.1%), while the lowest was at caliper 0.3 (69.3%, SD = 19.9%). Calipers 0.1 and 0.5 had comparable coverage rates of 71.2% (SD = 16.8%) and 70.1% (SD = 20.1%), respectively. The standard deviation increased slightly with larger calipers, indicating greater variability in matching success across datasets. Consistent coverage across calipers indicates that the 2D-PSM method remains effective at matching, regardless of caliper stringency, retaining a substantial portion of the treated cohort for subsequent causal analysis.

The performance advantage of 2D-PSM scaled systematically with dataset complexity. For the simplest synthetic scenarios (A-C, linear relationships only), 2D-PSM won or tied in 6 of 9 datasets when the caliper was set to 0.1. Loosening the caliper brought this number to 8 out of 9 for the 0.3 and 0.5 calipers. As complexity increased with the introduction of interaction terms, the 2D-PSM advantage became more pronounced, performing at least as well as the alternatives in all the most complex scenarios (E-G), except scenario F, which had more numerical parameters and a caliper of 0.1.

Covariate composition had minimal impact on 2D-PSM performance. In nearly all scenarios (balanced, more numerical, and more binary covariates), 2D-PSM either outperformed or matched the performance in 6 out of 7 cases across all calipers, except for caliper 0.1 with numerical and binary bias, where it was 5 out of 7. A similar analysis of the traditional PSM showed the highest success rate, 4 out of 7, for calipers 0.1 and 0.2, specifically with balanced covariate types.

Among the model combinations tested for 2D-PSM, pairing two LSVMs for categorical and numerical covariates was the most common choice, occurring 20 times out of 104. The next most-used combinations were LSVM with LR and LSVM with BNB, with 16 and 15 uses, respectively. Interestingly, across the RW datasets, no single combination was predominant, suggesting that different models may perform better in different cases. The top ten ML model combinations and the number of datasets for which they delivered the best results are presented in Table 4.

### Univariate balance assessment

Separate balance assessments for each covariate were conducted using ASMD and SDP metrics, with a threshold of 0.1. To evaluate PSM performance across all covariates in the dataset, we counted the number of covariates that were balanced after PSM. Overall, across all datasets, the percentage of covariates balanced with 2D-PSM never dropped below 75%. The count of balanced covariates was relatively evenly distributed across different datasets and caliper values. Furthermore, no significant differences were observed in the distributions between the traditional PSM results and 2D-PSM.

**Figure 1:**
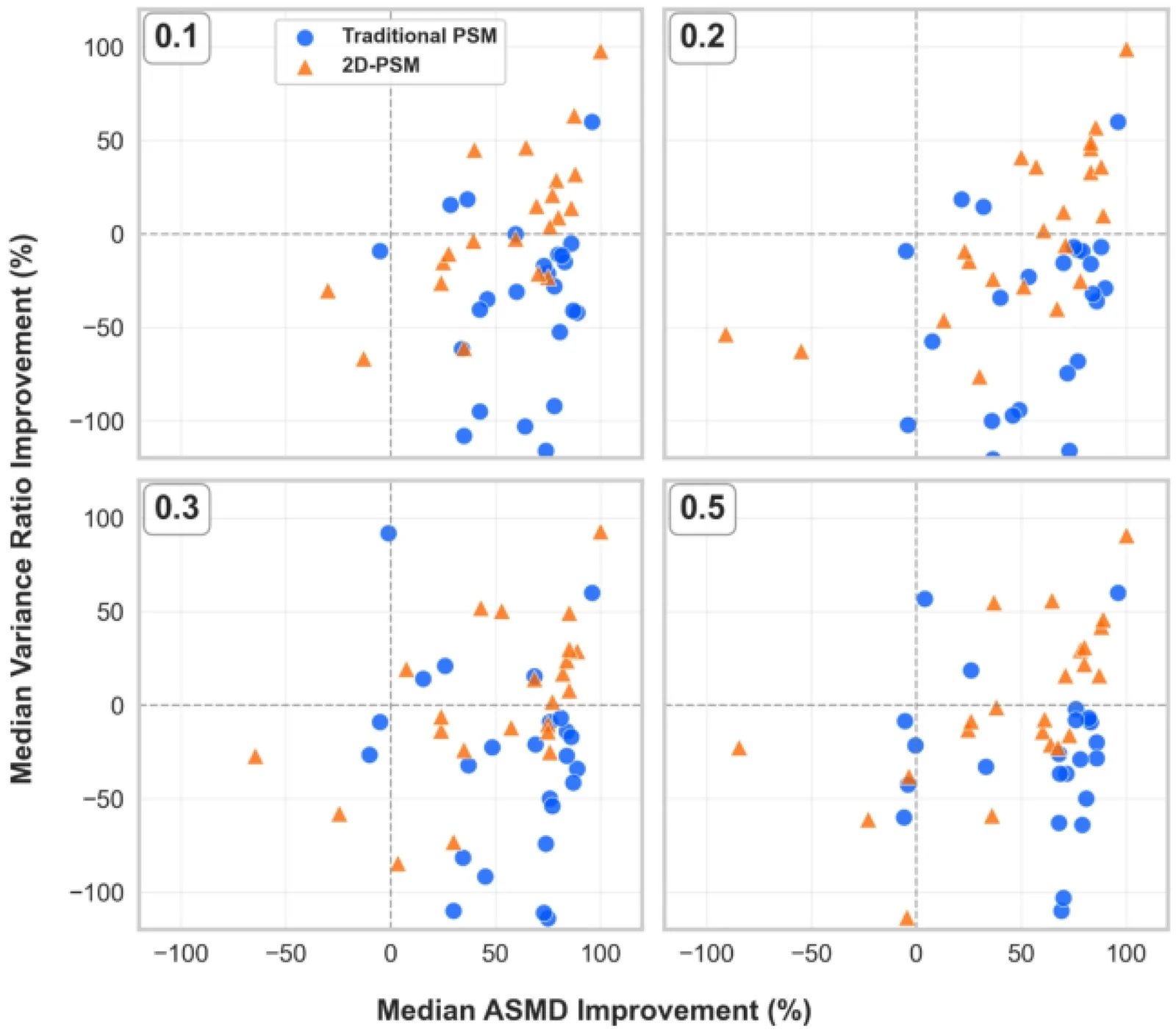
Median ASMD improvement (%) versus median variance ratio improvement (%) per dataset for Traditional PSM (circles) and 2D-PSM (triangles) across four caliper values (0.1, 0.2, 0.3, 0.5). Each point represents the median metric improvement across all covariates and models within a single dataset (n = 26 datasets per model type per panel). Dashed lines at zero separate improvement (positive values) from worsening (negative values) of covariate balance after matching.

While both 2D-PSM and traditional PSM typically improved ASMD for continuous covariates, we observed that VR frequently worsened despite ASMD improvements (Fig. 1). Both approaches show positive ASMD improvements across most datasets, but the traditional approach showed a substantially worse VR degradation. Figure 1 reveals that traditional PSM optimizes mean balance (ASMD) well but at the expense of distributional balance (VR). 2D-PSM sacrifices some ASMD performance to achieve a more balanced outcome across both metrics. Points in the upper-right quadrant (simultaneous improvement on both metrics) are predominantly 2D-PSM. The overall pattern remains remarkably stable across all four caliper values, suggesting that the relative advantage of 2D-PSM over traditional PSM in VR preservation is not an artifact of caliper selection but a structural property of the dual-model approach.

## Discussion

### Principal Findings and the 2D-PSM Framework

PSM methods are extensively used in medical and public health research, yet the conventional approach of condensing all covariate information into a single scalar score remains largely unchallenged (Rosenbaum and Rubin 1983; Cannas and Arpino 2019; Westreich et al. 2010). Previous studies have explored the performance of different ML classifiers for PS estimation, typically comparing them under a single-score framework using either simulated or RW data (Cannas and Arpino 2019; Setoguchi et al. 2008; Lee et al. 2010; Tian et al. 2018; Pirracchio and Carone 2018; McCaffrey et al. 2004). In this study, we introduced 2D-PSM, a method that decomposes the covariate space into categorical and numerical dimensions and matches in the two-dimensional PS space under an elliptical caliper constraint. Across 26 datasets (5 real-world EHR and 21 synthetic) and four caliper widths, 2D-PSM achieved superior or equivalent multivariate balance in 73.1% to 88.5% of conditions, with the advantage becoming more pronounced at moderate-to-large calipers and in the most complex treatment-assignment scenarios. These findings suggest that explicitly accounting for covariate heterogeneity during the matching stage can yield meaningful improvements in overall balance without sacrificing treated-cohort coverage.

Previous studies have also recognized the challenges posed by mixed covariate types for PS estimation. Brookhart et al. (2006) noted that variable selection for PS models entails inherent trade-offs, and Austin (2008) documented widespread shortcomings in the implementation and reporting of PSM in applied medical research. Yet to our knowledge, no prior approach has attempted to address the fundamental single-score compression problem by explicitly decomposing the matching process across covariate types. The 2D-PSM framework departs from this convention, and the consistency of its advantages across diverse datasets and conditions suggests that the decomposition captures a genuine structural feature of mixed-type covariate data.

The decomposition underlying 2D-PSM offers three key advantages over single-score approaches. First, it enables dimension-specific balance optimization. By estimating separate PSs for categorical and numerical covariates, the method creates a two-dimensional space where each unit is represented by a point with two PS values as coordinates, allowing researchers to independently assess and optimize balance in each dimension. The conventional single-score approach compresses heterogeneous covariate information into a unidimensional summary, a process in which balance for one covariate type may come at the expense of another (Franklin et al. 2014; Austin 2009). The two-dimensional representation makes these tradeoffs explicit and adjustable through the elliptical caliper parameters, providing researchers with a transparent view of the balance landscape across covariate types.

Second, 2D-PSM allows dimension-specific model selection. Different ML algorithms exhibit different strengths depending on whether covariates are categorical or continuous (Cannas and Arpino 2019). For example, BNB performs optimally with binary features but poorly with continuous features, a pattern well documented in the classification literature and confirmed in our results. Conversely, models such as LSVM and XGB handle continuous variables well but may not exploit the discrete structure of categorical data as effectively as specialized classifiers. The 2D-PSM framework permits fitting models best suited to each data type independently, rather than forcing a single classifier to handle both types simultaneously. This may partly explain why LSVM emerged as the most frequently best-performing model pairing (20 of 104 conditions). Its ability to learn linear decision boundaries generalizes well across both binary and continuous feature spaces. Notably, no single model combination dominated across all RW datasets, reinforcing prior findings that model performance depends on dataset characteristics (Cannas and Arpino 2019; Setoguchi et al. 2008; Lee et al. 2010; McCaffrey et al. 2004) and suggesting that researchers using 2D-PSM should evaluate multiple combinations, as is already recommended for traditional PSM (Westreich et al. 2010).

Third, the elliptical caliper constraint offers a more flexible matching geometry than rectangular or independent per-dimension constraints. The elliptical boundary, defined by (1), captures the joint distribution of PS differences across both dimensions, allowing compensatory balance: if two individuals differ more in one dimension, they can still be matched, provided they differ correspondingly less in the other. This property is particularly relevant in clinical datasets where categorical factors (e.g., race, sex) and continuous measurements (e.g., age, weight) may contribute differentially to treatment assignment, and strict univariate caliper constraints could unnecessarily exclude otherwise well-matched pairs. Unlike traditional PSM, where a single caliper constrains matching along a single axis, the elliptical caliper provides a natural way to express researchers' preferences about the relative importance of balance in categorical versus numerical covariates by adjusting the ellipse's semi-axis lengths.

### The ASMD-Variance Ratio Tradeoff

Another finding of this study concerns the relationship between ASMD and VR after matching. While both 2D-PSM and traditional PSM improved ASMD for continuous covariates, we observed that VR frequently worsened despite these improvements in ASMD (Fig. 1). Traditional PSM achieved larger ASMD reductions (approximately 70% median improvement) but at the cost of substantial VR degradation (approximately – 27% to −33% median change). By contrast, 2D-PSM produced more moderate ASMD improvements (approximately 55%) but with substantially less VR degradation (approximately – 9%). This pattern was remarkably stable across all four caliper values, indicating that the relative advantage of 2D-PSM in VR preservation is a structural property of the dual-model approach rather than an artifact of caliper selection.

This observation has important implications for downstream causal inference, which has received insufficient attention in the PSM literature. ASMD, the most widely reported balance diagnostic (Austin 2008; Franklin et al. 2014), captures only first-moment balance – it measures whether the means of covariates are similar between treated and control groups (Franklin et al. 2014; Austin 2009). However, two distributions can have identical means while differing substantially in their spread, shape, and tails. The variance ratio, which Rubin (2001) recommended keeping between 0.5 and 2.0 (with values between 0.8 and 1.25 considered ideal), captures second-moment balance and reflects whether matched groups have comparable dispersion across the covariate space. When VR worsens substantially, several consequences follow for the estimation of treatment effects. Many causal inference methods applied to matched samples (e.g., regression adjustment, weighted estimators) assume similar distributional properties across groups, and unequal variances can bias estimates of the average treatment effect even when means are perfectly balanced (Rubin 2001; Zhang et al. 2019). Unequal variances directly affect the precision of estimated treatment effects, potentially producing incorrect confidence intervals and p-values (Franklin et al. 2014). Additionally, a false sense of balance may arise: a researcher relying solely on the conventional ASMD < 0.1 threshold might conclude that matching "worked" while the matched cohorts retain fundamentally different distributional properties (Austin 2009; Rubin 2001; Zhang et al. 2019).

This concern is not merely theoretical. Lee et al. (2010) observed that logistic regression often yielded the lowest ASMD values yet produced significant bias in the estimated treatment effect, whereas boosted CART models that did not consistently yield the lowest ASMD frequently led to greater bias reduction. Their finding implies that the relationship between the univariate mean balance and treatment-effect bias is not straightforward, particularly when distributional properties beyond the first moment are disrupted by matching. Our results extend this observation by showing that the ASMD-VR divergence is systematic and predictable: traditional PSM consistently favors mean balance at the expense of distributional balance, while 2D-PSM achieves a more balanced outcome across both metrics. Specifically, the upper-right quadrant of Fig. 1, representing simultaneous improvement in both ASMD and VR, was predominantly populated by 2D-PSM results across all caliper settings. In practical terms, when matching quality is evaluated solely on mean balance, traditional PSM appears superior to 2D-PSM. However, when distributional balance is important, 2D-PSM yields a substantially better overall covariate balance profile by simultaneously constraining balance across both dimensions.

It is worth noting that the mean-variance tradeoff was observed for both traditional PSM and 2D-PSM, which suggests that this phenomenon may be intrinsic to greedy nearest-neighbor matching rather than specific to the dimensional matching strategy. Greedy algorithms, which sequentially select the best available match for each treated individual, can progressively deplete the control pool in ways that distort variance even as mean balance is maintained (Austin 2014). However, 2D-PSM mitigates the severity of this tradeoff. By constraining matching in two dimensions simultaneously, the elliptical caliper prevents the optimizer from aggressively "pulling" means together while drawing controls from disparate regions of the covariate space. These findings highlight the importance of evaluating both ASMD and VR jointly when assessing match quality, precisely the diagnostic approach recommended by Austin (2009) and Rubin (2001), yet often neglected in applied research (Austin 2008).

### Caliper Effects and Dataset Complexity

The progressive improvement of 2D-PSM relative to traditional PSM with increasing caliper width (from 73.1% at 0.1×IQR to 88.5% at 0.5×IQR) is straightforward to interpret. At the most restrictive caliper, both methods are heavily constrained: only very close matches in PS space are permitted, and the advantage of matching in two dimensions rather than one is limited because the matching region is narrow regardless of its geometry. As calipers widen, the elliptical matching region in 2D-PSM expands in a way that allows the method to exploit compensatory balance between dimensions – a degree of freedom that single-score PSM fundamentally lacks. Importantly, this advantage did not come at the cost of reduced matching rates: treated-cohort coverage remained comparable across caliper values (approximately 70–73%), indicating that the 2D method retains a substantial portion of the treated cohort for analysis regardless of caliper stringency. This coverage stability is notable given that the elliptical constraint imposes a fundamentally different geometric boundary than the interval constraint used in traditional caliper matching, and confirms that the 2D approach does not introduce a coverage penalty.

The performance advantage of 2D-PSM also scaled systematically with treatment-assignment complexity. For simple linear scenarios (A-C), 2D-PSM performed comparably to or slightly better than traditional PSM. As complexity increased through the introduction of interaction terms (scenarios D-G), the 2D-PSM advantage became more pronounced, achieving at least parity with traditional PSM in all of the most complex scenarios. This scaling is expected: when treatment assignment involves complex interactions between categorical and numerical covariates, a single model fitting all covariates simultaneously may struggle to capture the distinct contributions of each covariate type, whereas the decomposed approach can model each dimension's relationship to treatment assignment more accurately. Covariate composition (balanced, more numerical, or more binary) had minimal impact on 2D-PSM performance, with the method achieving wins or ties in 6 of 7 scenarios across nearly all compositions and caliper values. By contrast, traditional PSM showed its highest success rate of only 4 out of 7 scenarios, specifically with balanced covariate types and tighter calipers.

Among the ML models evaluated, LSVM emerged as the most frequent top-performing combination (20 of 104 conditions), followed by LSVM paired with LR (16 conditions) and LSVM with BNB (15 conditions). This finding aligns with prior literature showing that linear models often perform competitively for PS estimation (Cannas and Arpino 2019; Setoguchi et al. 2008; Westreich et al. 2010) as the primary goal is to achieve covariate balance rather than maximize predictive accuracy. The lack of a single dominant model combination across RW datasets underscores the importance of dataset-specific tuning in the 2D-PSM framework, consistent with recommendations for traditional PSM (Cannas and Arpino 2019; Lee et al. 2010). In practice, we recommend that researchers evaluate several model combinations and select the one that achieves the best multivariate balance for their specific dataset, analogous to the model selection process already standard in traditional PSM workflows.

### Limitations and Future Directions

Several limitations of this study should be acknowledged. We used only 1:1 greedy nearest-neighbor matching. Other methods, such as optimal matching (Austin 2014), variable-ratio matching (Ming and Rosenbaum 2000), full matching, and caliper matching with replacement (Rassen et al. 2012), may interact differently with the two-dimensional framework and could either enhance or reduce its benefits. Exploring these options is an important future step. We tested five ML classifiers; however, approaches such as neural networks, Super Learner ensembles (Pirracchio and Carone 2018; Neugebauer et al. 2016), and covariate-balancing PSs have not yet been evaluated within the 2D-PSM framework. Third, although our synthetic data varied systematically in treatment-assignment complexity and covariate makeup, it cannot fully mimic all features of real clinical data, such as missing data, time-varying confounders, or unmeasured confounding. Additionally, the 2D-PSM framework incurs an additional computational cost because it requires fitting two models per dimension, but this cost is relatively minor relative to the overall matching process. Another limitation is the fixed caliper widths we tested based on fractions of the IQR. Using data-driven or adaptive caliper strategies could further enhance performance.

Perhaps the most important limitation is that this study evaluated covariate balance rather than treatment effect, which is the ultimate goal of PS methods. While improved covariate balance is a necessary condition for unbiased treatment effect estimation, it is not sufficient, as residual confounding from unmeasured covariates cannot be addressed by any matching method (Rosenbaum and Rubin 1983; Lee et al. 2010). Future work should evaluate 2D-PSM using datasets with known true treatment effects to directly assess whether the observed balance improvements translate into reduced bias in estimated causal effects. Additionally, developing formal caliper selection procedures tailored to the two-dimensional setting would provide practical guidance for applied researchers, as current caliper recommendations (e.g., Austin's 0.2 standard deviations of the logit of the PS; Austin 2011) were derived for the single-score context and may not generalize directly to elliptical calipers. Finally, extending the framework beyond two dimensions (for example, by separating ordinal, nominal, and continuous covariates into three or more PS dimensions) represents a conceptually appealing generalization that warrants investigation, though it would require addressing the increased computational and geometric complexity of higher-dimensional matching spaces.

## Conclusion

The optimal PSM strategy depends on dataset complexity, confounder types, and the chosen balance metric. Our 2D-PSM approach demonstrated superior multivariate balance across most scenarios, particularly for datasets with complex categorical-continuous variable interactions. The discrepancy between univariate and multivariate assessment highlights the importance of comprehensive balance evaluation. Future work will explore alternative matching algorithms (optimal, genetic) and assess downstream effects on treatment effect estimation.

## Figures and Tables

**Table 1:** Real-World Dataset Characteristics

Dataset	Total Patients	Treated Patients (%)	Total Covariates (Numerical: Categorical)	Clinical Context
RWD1	22,905	295 (1.3%)	18(11:7)	End-stage renal disease with extensive comorbidities
RWD2	13,911	2,097 (15.1%)	3(2:1)	Menopausal burn patients: demographic confounders
RWD3	61,926	25,829 (41.7%)	7(5:2)	Reconstructive surgery: the largest sample, balanced treatment
RWD4	460	360 (78.3%)	14(13:1)	Pulmonary embolism: small N, high treatment prevalence
RWD5	809	153 (18.9%)	5(3:2)	Renal transplant recipients: an obese subpopulation

**Table 2:** Treatment Assignment Scenarios in Synthetic Datasets

Scenario	Linear covariates	Quadratic covariates	Interaction pairs	Description
A	20	n/a	n/a	Linear only
B	20	2	n/a	Low nonlinearity
C	20	4	n/a	Moderate nonlinearity
D	20	n/a	5	Moderate interactions
E	20	n/a	10	High interactions
F	20	2	5	Combined moderate
G	20	4	10	Maximum complexity

**Table 3:** Summary Performance of 2D-PSM

Caliper,	Real-World Datasets			Synthetic Dataset			Wins and Ties Rate, %
×IQR	Wins	Ties	Total	Wins	Ties	Total	
0.1	2	1	5	11	5	21	73.1
0.2	3	0	5	10	8	21	80.8
0.3	2	0	5	15	4	21	80.8
0.5	2	1	5	20	0	21	88.5

**Table 4:** The Top 10 Model Combinations.

Model combinations (categorical x numerical)	Number of datasets
Linear SVM x Linear SVM	20
Linear SVM x Logistic Regression	16
Bernoulli NB x Linear SVM	15
Logistic Regression x Linear SVM	11
Logistic Regression x Logistic Regression	10
Random Forest x Linear SVM	10
Bernoulli NB x Logistic Regression	9
XGBoost x Linear SVM	9
XGBoost x Logistic Regression	6
Random Forest x Logistic Regression	5

## Data Availability

The datasets generated and analyzed during the current study are available in figshare.com repository, https://doi.org/10.6084/m9.figshare.28871060

## References

[1] Austin P.C.: A critical appraisal of propensity-score matching in the medical literature between 1996 and 2003. Stat. Med. 27(12), 2037–2049 (2008). 10.1002/sim.3150 18038446

[2] Austin P.C.: Balance diagnostics for comparing the distribution of baseline covariates between treatment groups in propensity-score matched samples. Stat. Med. 28(25), 3083–3107 (2009) 19757444 10.1002/sim.3697 PMC3472075

[3] Austin P.C.: Optimal caliper widths for propensity-score matching when estimating differences in means and differences in proportions in observational studies. Pharm. Stat. 10(2), 150–161 (2011). 10.1002/pst.433 20925139 PMC3120982

[4] Austin P.C.: A comparison of 12 algorithms for matching on the propensity score. Stat. Med. 33(6), 1057–1069 (2014). 10.1002/sim.6004 24123228 PMC4285163

[5] Austin P.C., Yu A.Y.X., Vyas M.V., Kapral M.K.: Applying Propensity Score Methods in Clinical Research in Neurology. Neurology. 97(18), 856–863 (2021). 10.1212/WNL.0000000000012777 34504033 PMC8610625

[6] Brookhart M.A., Schneeweiss S., Rothman K.J., Glynn R.J., Avorn J., Stürmer T.: Variable selection for propensity score models. Am. J. Epidemiol. 163(12), 1149–1156 (2006) 16624967 10.1093/aje/kwj149 PMC1513192

[7] Cannas M., Arpino B.: A comparison of machine learning algorithms and covariate balance measures for propensity score matching and weighting. Biom. J. 61(4), 1049–1072 (2019) 31090108 10.1002/bimj.201800132

[8] Chen J.W., Maldonado D.R., Kowalski B.L., : Best practice guidelines for propensity score methods in medical research: consideration on theory, implementation, and reporting. A review. Arthroscopy: J. Arthroscopic Relat. Surg. 38(2), 632–642 (2022) 10.1016/j.arthro.2021.06.037 34547404

[9] D’Agostino R.B.: Propensity score methods for bias reduction in the comparison of a treatment to a non-randomized control group. Statist Med. 17(19), 2265–2281 (1998). 10.1002/(SICI)1097-0258(19981015)17:19<2265::AID-SIM918>3.0.CO;2-B 9802183

[10] Dongur L., Samman Y., Golovko G., : Cancer incidence following bariatric surgery in renal transplant recipients: a retrospective multi-center analysis. Surgery for Obesity and Related Diseases. Published online 2024. 10.1016/j.soard.2024.06.010 PMC12922797 39138043

[11] Drake C.: Effects of misspecification of the propensity score on estimators of treatment effect. Biometrics Published online 1993:1231–1236

[12] Franchetti Y.: Use of Propensity Scoring and Its Application to Real-World Data: Advantages, Disadvantages, and Methodological Objectives Explained to Researchers Without Using Mathematical Equations. J. Clin. Pharma. 62(3), 304–319 (2022). 10.1002/jcph.1989 34671990

[13] Franklin J.M., Rassen J.A., Ackermann D., Bartels D.B., Schneeweiss S.: Metrics for covariate balance in cohort studies of causal effects. Stat. Med. 33(10), 1685–1699 (2014) 24323618 10.1002/sim.6058

[14] Gharibzadeh S., Mansournia M.A., Rahimiforoushani A., : Comparing different propensity score estimation methods for estimating the marginal causal effect through standardization to propensity scores. Commun. Stat. - Simul. Comput. 47(4), 964–976 (2018). 10.1080/03610918.2017.1300267

[15] Gu X.S., Rosenbaum P.R.: Comparison of Multivariate Matching Methods: Structures, Distances, and Algorithms. J. Comput. Graphical Stat. 2(4), 405–420 (1993). 10.1080/10618600.1993.10474623

[16] Guo S., Fraser M., Chen Q.: Propensity Score Analysis: Recent Debate and Discussion. J. Soc. Social Work Res. 11(3), 463–482 (2020). 10.1086/711393

[17] Iacus S.M., King G., Porro G.: Multivariate matching methods that are monotonic imbalance bounding. J. Am. Stat. Assoc. 106(493), 345–361 (2011)

[18] Kane L.T., Fang T., Galetta M.S., : Propensity score matching: a statistical method. Clin. spine Surg. 33(3), 120–122 (2020) 31913173 10.1097/BSD.0000000000000932

[19] Langworthy B., Wu Y., Wang M.: An overview of propensity score matching methods for clustered data. Stat. Methods Med. Res. 32(4), 641–655 (2023). 10.1177/09622802221133556 36426585 PMC10119899

[20] Lee B.K., Lessler J., Stuart E.A.: Improving propensity score weighting using machine learning. Stat. Med. 29(3), 337–346 (2010) 19960510 10.1002/sim.3782 PMC2807890

[21] McCaffrey D.F., Ridgeway G., Morral A.R.: Propensity score estimation with boosted regression for evaluating causal effects in observational studies. Psychol. Methods. 9(4), 403 (2004) 15598095 10.1037/1082-989X.9.4.403

[22] Ming K., Rosenbaum P.R.: Substantial Gains in Bias Reduction from Matching with a Variable Number of Controls. Biometrics. 56(1), 118–124 (2000). 10.1111/j.0006-341X.2000.00118.x 10783785

[23] Neugebauer R., Van Der Schmittdiel J.A.: Int. J. Biostatistics. 12(1), 131–155 (2016). 10.1515/ijb-2015-0028 A Case Study of the Impact of Data-Adaptive Versus Model-Based Estimation of the Propensity Scores on Causal Inferences from Three Inverse Probability Weighting Estimators PMC6052862 27227720

[24] Pirracchio R., Carone M.: The Balance Super Learner: A robust adaptation of the Super Learner to improve estimation of the average treatment effect in the treated based on propensity score matching. Stat. Methods Med. Res. 27(8), 2504–2518 (2018). 10.1177/0962280216682055 28339317

[25] Rassen J.A., Shelat A.A., Myers J., Glynn R.J., Rothman K.J., Schneeweiss S.: One-to-many propensity score matching in cohort studies. Pharmacoepidemiol. Drug Saf. 21(S2), 69–80 (2012). 10.1002/pds.3263 22552982

[26] Rosenbaum P.R., Rubin D.B.: The central role of the propensity score in observational studies for causal effects. Biometrika. 70(1), 41–55 (1983)

[27] Rosenbaum P.R., Rubin D.B.: Reducing Bias in Observational Studies Using Subclassification on the Propensity Score. J. Am. Stat. Assoc. 79(387), 516–524 (1984). 10.1080/01621459.1984.10478078

[28] Rosenbaum P.R., Rubin D.B., Constructing a Control Group Using Multivariate Matched Sampling Methods That Incorporate the Propensity Score: Am. Stat. 39(1), 33–38 (1985). 10.1080/00031305.1985.10479383

[29] Rubin D.B.: Using propensity scores to help design observational studies: application to the tobacco litigation. Health Serv. Outcomes Res. Method. 2(3–4), 169–188 (2001). 10.1023/A:1020363010465

[30] Setoguchi S., Schneeweiss S., Brookhart M.A., Glynn R.J., Cook E.F.: Evaluating uses of data mining techniques in propensity score estimation: a simulation study. Pharmacoepidemiol. Drug Saf. 17(6), 546–555 (2008) 18311848 10.1002/pds.1555 PMC2905676

[31] Song J., Golovko G., Botnar K., El Ayadi A., Vincent K.L., Wolf S.E.: Estrogen Treatment Lowers the Risk of Complications in Menopausal Women with Mild Burn Injury. Medicina. 61(2), 300 (2025). 10.3390/medicina61020300 40005417 PMC11857297

[32] Tian Y., Schuemie M.J., Suchard M.A.: Evaluating large-scale propensity score performance through real-world and synthetic data experiments. Int. J. Epidemiol. 47(6), 2005–2014 (2018) 29939268 10.1093/ije/dyy120 PMC6280944

[33] Tu C.: Comparison of various machine learning algorithms for estimating generalized propensity score. J. Stat. Comput. Simul. 89(4), 708–719 (2019). 10.1080/00949655.2019.1571059

[34] Westreich D., Lessler J., Funk M.J.: Propensity score estimation: machine learning and classification methods as alternatives to logistic regression. J. Clin. Epidemiol. 63(8), 826 (2010) 20630332 10.1016/j.jclinepi.2009.11.020 PMC2907172

[35] Zhang Z., Kim H.J., Lonjon G., Zhu Y.: Balance diagnostics after propensity score matching. Annals Translational Med. 7(1), 16 (2019). 10.21037/atm.2018.12.10 PMC6351359 30788363

[36] Zhao P., Su X., Ge T., Fan J.: Propensity score and proximity matching using random forest. Contemp. Clin. Trials. 47, 85–92 (2016). 10.1016/j.cct.2015.12.012 26706666 PMC4818178

